# Comparison of physical workload and physical work capacity among municipality cleaners in Shiraz to determine number of workers needed to counterbalance physical workload

**DOI:** 10.1186/s13102-022-00476-4

**Published:** 2022-05-07

**Authors:** Farnaz Bagherifard, Hadi Daneshmandi, Mansour Ziaei, Haleh Ghaem, Ruhollah Khoshbakht, Omid Jaberi, Alireza Choobineh

**Affiliations:** 1 Department of Occupational Health Engineering, School of Health, Shiraz University of Medical Sciences, Shiraz, Iran; 2 Research Center for Health Sciences, Institute of Health, Shiraz University of Medical Sciences, PO Box: 71645-111, Shiraz, Iran; 3 School of Health and Nutrition, Bushehr University of Medical Sciences, Bushehr, Iran; 4 Department of Epidemiology, School of Health, Shiraz University of Medical Sciences, Shiraz, Iran; 5 Health and Safety Executive Unit, Shiraz Waste Management Organization, Shiraz, Iran

**Keywords:** Cleaners, Maximum aerobic capacity, Physical workload, Physical work capacity

## Abstract

**Background:**

Assessing physical workload is the most important step in deciding whether a workload is high and adopting appropriate control strategies to reduce physical workload. This study aimed to compare physical workload and Physical Work Capacity (PWC) among municipality cleaners in Shiraz to determine the number of workers needed to counterbalance physical workload.

**Methods:**

The present cross-sectional study was performed on 97 municipality cleaners in Shiraz. In the first step, the participants' maximum aerobic capacity (VO_2_-max) was estimated in the laboratory using an ergometer bicycle and the Young Men’s Christian Association (YMCA) protocol, based on which the PWC was estimated. Secondly, energy expenditure and heart rate during work were measured using a POLAR400 device in an eight-hour shift. At the end of the work shift, the workers’ perceived physical exertion was assessed using a Rating of Perceived Exertion 6–20 (RPE 6–20) Borg scale. In the final stage, the physical workload was assessed based on the results of the two steps.

**Results:**

The mean VO_2_-max of the cleaners and PWC were estimated to be 2.6 ± 0.66 l min^−1^ and 4.3 ± 1.088 kcal min^−1^, respectively. The average energy consumed during work was 4.122 ± 1.016 kcal min^−1^. The overall results of this study showed that physical workload was greater than PWC in 46% of the municipality cleaners. In addition, it was found that 12.45% workforce was required to be added to the street cleaners of Shiraz municipality to reduce the physiological workload on the employed workforce.

**Conclusions:**

With respect to the high level of physical activity in a significant proportion of the cleaners, measures such as increasing the workforce are suggested.

## Background

Street cleaners have an important responsibility for eliminating harmful waste and protecting public health and sanitation [1]. Due to and required by their responsibility, this group of workers is potentially exposed to a wide range of adverse factors and occupational hazards [2–8]. The hazards result from physical, chemical, and biological exposures but may also be due to physiological and psychological burdens or inadequate safety aspects. The most commonly reported work-related complaints are musculoskeletal and respiratory disorders, cuts, slips, and road traffic accidents. In developing countries, street cleaners seem to be still heavily exposed to dust, and in most cases, no suitable protective measures are available [9, 10]. The cleanliness job consists of various tasks, including sweeping while standing for a long time, bending to collect waste, pulling and pushing waste collection containers, and manually carrying objects (usually waste or waste collection containers). Due to work difficulty and walking over long distances, the physical workload is one of the most important ergonomic risk factors amongst cleaners. Therefore, the job of cleaners is often considered to be difficult and excruciating [4, 11–13].

If the physical workload exceeds one’s capacity to perform it, there are various complications and consequences, including musculoskeletal disorders and impaired cardiovascular function. In addition, overload causes excessive fatigue and burnout, decreased efficiency and job satisfaction, increased workplace complaints, increased absenteeism, reduced cognitive performance, and increased likelihood of human errors or accidents [14–16]. Therefore, to maintain the health of workers and prevent premature burnout of the workforce and other consequences of increased workload, it is necessary to maintain a reasonable balance between the energy required to perform the work (physical work demand) and the Physical Work Capacity (PWC) of the worker [14, 17]. PWC represents the highest amount of energy a person can consume during an eight-hour work shift without damaging one’s health [18, 19]. Bonjer proposed 33% of the maximum aerobic capacity (VO_2_-max) as an acceptable threshold for energy consumption, which is still accepted by work physiology researchers today [18, 19]. Hence, to assess physical workload, one must first determine the individual’s PWC and compare it to the energy consumed during the work to assess one’s physical workload [19]. The physical workload is the measurable portion of physical resources expended when performing a given task (manual lifting and carrying, repetitive work, and other physical strain). It is affected by various factors, including the nature of work, training, motivation, and environmental factors [20].

In recent decades, there has been a great deal of research on PWC and the workload of industrial workers and public service workers in different societies [14, 21–29]. A review of past studies has shown that despite the importance of assessing physical workload, an issue of such importance has not yet been addressed in Iranian cleaners. Indeed, the studies carried out abroad on assessing the physical workload of municipality workers have focused on waste collection workers or domestic cleaning workers [12, 24, 30–33]. As a result, studies on assessing the physical workload of the cleaner population are scarce [11, 13]. The present study was undertaken among municipality cleaners in Shiraz with the following objectives:

- Determining the VO_2_-max and PWC
- Assessing the physical workload by determining energy expenditure, heart rate, and Rating of Perceived Exertion 6–20 (RPE 6–20) Borg scale during work
- Determining the association between “PWC”, and “energy expenditure during work” with demographic/occupational variables
- Comparison of “energy expenditure during work” and “PWC”, and determining the number of municipality cleaners needed to reduce the physical workload

## Methods

This cross-sectional study was carried out on 97 male municipality cleaners in Shiraz. The samples were selected from ten districts of Shiraz municipality using random cluster sampling. To do so, the number of samples in different areas was first determined based on the number of cleaners working in that area, and then, the samples in each area were randomly selected. The inclusion criteria were signing the informed consent to participate in the study, having at least one year of work experience, and not having a history of respiratory and cardiovascular diseases.

This study was conducted in three phases as follows:

*Phase 1:* The workers’ VO_2_-max was estimated during the first phase. At this phase, before the experiment, a written consent form and a demographic questionnaire were provided to the individuals. After explaining how to complete the questionnaire and the researchers’ ethical obligations to the cleaners, they were asked to participate in the study. The questionnaire consisted of two parts. The first part was completed through face-to-face interviews, and the second part by measuring the required parameters by the researcher. The first part of the questionnaire encompassed variables, such as age, work experience, marital status, education level, shift work, specific illnesses, smoking status, exercise, working hours during a shift, fatigue at work, and taking medicines. The second part of the questionnaire included anthropometric and physiological measurements. A stadiometer (made in Iran) and a scale (Beurer, made in Germany) were used to measure height and weight, respectively.

In this phase (phase 1), the VO_2_-max of the individuals was assessed by the MONARK (Ergomedic 839 E, made in Sweden) ergometer bicycle in accordance with the Young Men’s Christian Association (YMCA) protocol [34] at the ergonomics laboratory of School of Health, Shiraz University of Medical Sciences, southwest of Iran.

In this method (YMCA), settings for each person, including first name, last name, gender, and height and weight values were first entered into the software. Then, the heart rate monitor was placed on the participants' chests. The ‘pedal cadence’ and ‘workload’ adjustments were made for each individual in the next step. For this purpose, a pedal cadence of 50 revolutions per minute (rpm) was considered. The YMCA is a popular protocol with a multistage format (each stage lasts for three minutes). Thus, the total test may last from 6 to 12 min. The first workload (first stage) is 150 kp m min^−1^ for everyone. In later stages, the workload increases based on the heart rate of the previous stage. After completing the test, the individuals’ VO_2_-max was calculated using the ergocycle software [34]. It should be noted that heart rate was measured using the chest belt Polar T34 [34].

After estimating VO_2_-max, the PWC was calculated. Since about five kcal of energy is released per liter of oxygen consumed, the measured VO_2_-max was initially multiplied by five and 33% of it was considered as PWC (kcal min^−1^) [19].

*Phase 2:* In the second phase of the study, the physical workload of the cleaners was measured while working in the field. To this end, energy expenditure and heart rate were measured in an eight-hour work shift using the Polar S400 heart rate monitor (made in Finland) [35, 36]. At the end of the work shift, the perceived physical exertion of the workers was assessed using a RPE 6–20 Borg scale [37]. RPE 6–20 is a tool for measuring an individual’s effort and exertion during physical work. In its simplest terms, it provides a measure of how hard it feels that the body is working based on the physical sensations that the subject experiences, including increased heart rate, increased respiration or breathing rate, increased sweating, and muscle fatigue. The unusual scaling, ranging not from 0 to 20 but from 6 to 20, is related to the high correlation between the scale and heart rate. Thus, a Borg RPE scale of 6 corresponds to a heart rate of 60 beats per minute (bpm) [37]. The validation of the Persian version of the RPE 6–20 has been examined by Daneshmandi et al. (r = 0.847) [38].

*Phase 3:* In the final phase, each participant’s PWC was compared to energy expenditure at work. Then, the participants were divided into two groups as follows:

- People whose energy expenditure while doing the work was lower than their PWC (did not experience physiological fatigue)
- People whose energy expenditure while working was higher than their PWC (experienced physiological fatigue).

The findings of this section revealed the shortage of workforce for carrying out the job. The number of workers needed to reduce the physical workload was calculated via Eq. 1.

1 $${\text{Number }}\,{\text{of}}\,{\text{workers}}\,{\text{required = }}\frac{{\sum \left( {\text{EE}} - {\text{PWC}} \right)\,{\text{for}}\,{\text{workers}}\,{\text{whose}}\,{\text{energy}}\,{\text{expenditure}}\,{\text{was}}\,{\text{higher}}\,{\text{than}}\,{\text{their}}\,{\text{PWC }}\,{\text{in}}\,{\text{a }}\,{\text{shift}}\,{(480}\,{\text{min) }}}}{{{\text{Average}}\,{\text{PWC}}\,{\text{for}}\,{\text{workers}}\,{\text{whose}}\,{\text{energy}}\,{\text{expenditure}}\,{\text{was}}\,{\text{higher}}\,{\text{than }}\,{\text{their}}\,{\text{PWC}}\,{\text{in}}\,{\text{a}}\,{\text{shift}}\,{(480 }\,{\text{min)}}}}$$

EE = Energy expenditure (kcal min^−1^), PWC = Physical Work Capacity (kcal min^−1^).

Afterward, the percentage of cleaners required to be added to each municipality zone was calculated via Eq. 2.

2 $${\text{Percentage}}\,{\text{of}}\,{\text{cleaners}}\,{\text{required}}\,{\text{to}}\,{\text{be}}\,{\text{added = }}\frac{{{\text{Number}}\,{\text{of}}\,{\text{cleaners}}\,{\text{who}}\,{\text{should }}\,{\text{be}}\,{\text{added }}\,{\text{to}}\,{\text{each }}\,{\text{municipality}}\,{\text{zone}}\,{\text{based}}\,{\text{on}}\,{\text{the}}\,{\text{sample }}\,{\text{size}}}}{{{\text{Number}}\,{\text{of}}\,{\text{participants}}\,{\text{selected}}\,{\text{from }}\,{\text{each}}\,{\text{municipality}}\,{\text{zone }}}}$$

### Statistical analysis

The Statistical Package for Social Sciences 16 (SPSS Inc., Chicago, IL, USA) was used to analyze the data. At first, Kolmogorov–Smirnov and Shapiro–Wilk tests were used to test the normality of the data. Since data had no normal distribution, non-parametric statistical analysis was used. Descriptive statistics (frequency, percentage, and mean ± standard deviation), Spearman's correlation coefficient, Linear regression, and Mann–Whitney U test were used to analyze the data. *P* < 0.05 was considered statistically significant.

### Ethical considerations

This study was approved by the local Ethics Committee of Shiraz University of Medical Sciences (Approval ID: IR.SUMS.REC.1397.997).

## Results

The demographic and occupational details of the study population have been presented in Table 1.

**Table 1:** Demographic and occupational details of the study population (n = 97)

Quantitative variable	Mean ± standard deviation	Minimum	Maximum
Age (years)	38.16 ± 7.65	25	65
Work experience (years)	9.99 ± 5.82	1	29
Exercise hours per week	0.99 ± 3	0	22
Height (cm)	172.90 ± 6.5	160	197
Weight (kg)	72.18 ± 12.68	47	107
BMI (kg m^−2^)	24.09 ± 3.74	16.9	34.16

Qualitative variable	Number	%
Marital status		
Single	2	2.1
Married	95	97.9
Education level		
Elementary school or lower	29	29.9
Middle school	41	42.3
High school and diploma	26	26.8
Higher education	1	0.1
Smoking		
Yes	25	25.77
No	72	74.23

BMI, Body Mass Index

The results of the descriptive data regarding VO_2_-max, PWC, energy expenditure, heart rate during work, and RPE 6–20 Borg scale have been presented in Table 2.

**Table 2:** Physiological parameters measured in the studied workers (n = 97)

Variables in the laboratory	Mean ± Standard deviation
VO_2_-max (l min^−1^)	2.60 ± 0.66
PWC (kcal min^−1^)	4.30 ± 1.09

Variables in the field	Mean ± Standard deviation
Energy expenditure during work (kcal min^−1^)	4.12 ± 1.02
Heart rate during work (bpm)	96.13 ± 18.09
RPE 6–20 Borg scale	16.60 ± 1.94

PWC, Physical Work Capacity; RPE, Rating of Perceived Exertion

First, the relationship between “PWC”, and “energy expenditure during work” with demographic/occupational variables (presented in Table 1) were investigated.

Based on the statistical analysis, there is no statistically significant difference between the “PWC” and “energy expenditure during work” (*P* = 0.226).

The Spearman's correlation coefficient and Mann–Whitney U test showed that among the demographic/occupational variables, there is only a statistically significant relationship between “BMI” and the “PWC” (r = 0.22, *P* = 0.045). The correlation between BMI and PWC is reflected in Eq. 3.

3 $${\text{PWC}} = \left( {0.0{57} \times {\text{BMI}}} \right) + {2}.{92}$$

PWC = Physical Work Capacity (kcal min^−1^), BMI = Body Mass Index (kg m^−2^).

The above statistical tests showed that there is no statistically significant relationship between “energy expenditure during work” with demographic/occupational variables (*P* > 0.05).

The comparison of PWC to energy expenditure showed that 46% of the cleaners (n = 45) exceeded their PWC. Then, we calculated “the number of workers needed to reduce the physical workload” and “the percentage of cleaners required to be added to each municipality zone” via Eqs. 1 and 2, respectively.

The number of workers needed to reduce the physical workload for each municipality zone has been presented in Table 3. Accordingly, 12.45% workforce was required to be added to all street cleaners of Shiraz municipality to reduce the physiological workload on the employed workforce.

**Table 3:** Number of workers needed to counterbalance physical workload for each municipality zone (n = 97)

Municipality zone	Total number of workers in each zone	Number of the participant in each zone ^a^	PWC (kcal min^−1^)		Energy expenditure (kcal min^−1^)		Number of participants with energy expenditure higher than PWC	Number of workers needed ^b^	Percentage of required workers ^c^
			M	SD	M	SD			
1	200	11	4.04	0.84	3.67	1.80	4	1.55	14.09
2	194	11	5.39	1.54	4.04	0.76	2	0.63	5.73
3	148	8	3.96	0.67	4.06	0.30	5	0.87	10.88
4	197	11	4.41	1.06	4.49	1.08	6	2.38	21.64
5	148	9	3.7	0.55	3.85	1.08	5	1.16	12.89
6	110	7	4.2	0.33	5	1.05	5	1.63	23.29
7	185	12	3.92	0.97	3.9	0.61	6	1.12	9.33
8	130	8	4.37	0.96	5.08	0.98	6	1.56	19.50
9	111	6	4.90	1.77	3.67	1.80	2	0.29	4.83
10	134	8	4.45	0.78	3.84	0.30	1	0.26	3.25
11	97	6	3.79	0.92	3.72	0.184	3	0.62	10.33
Total	1654	97	4.3	1.09	4.12	1.01	45	12.08	12.45

c = (b ÷ a) × 100

PWC, Physical Work Capacity

**Fig. 1:**
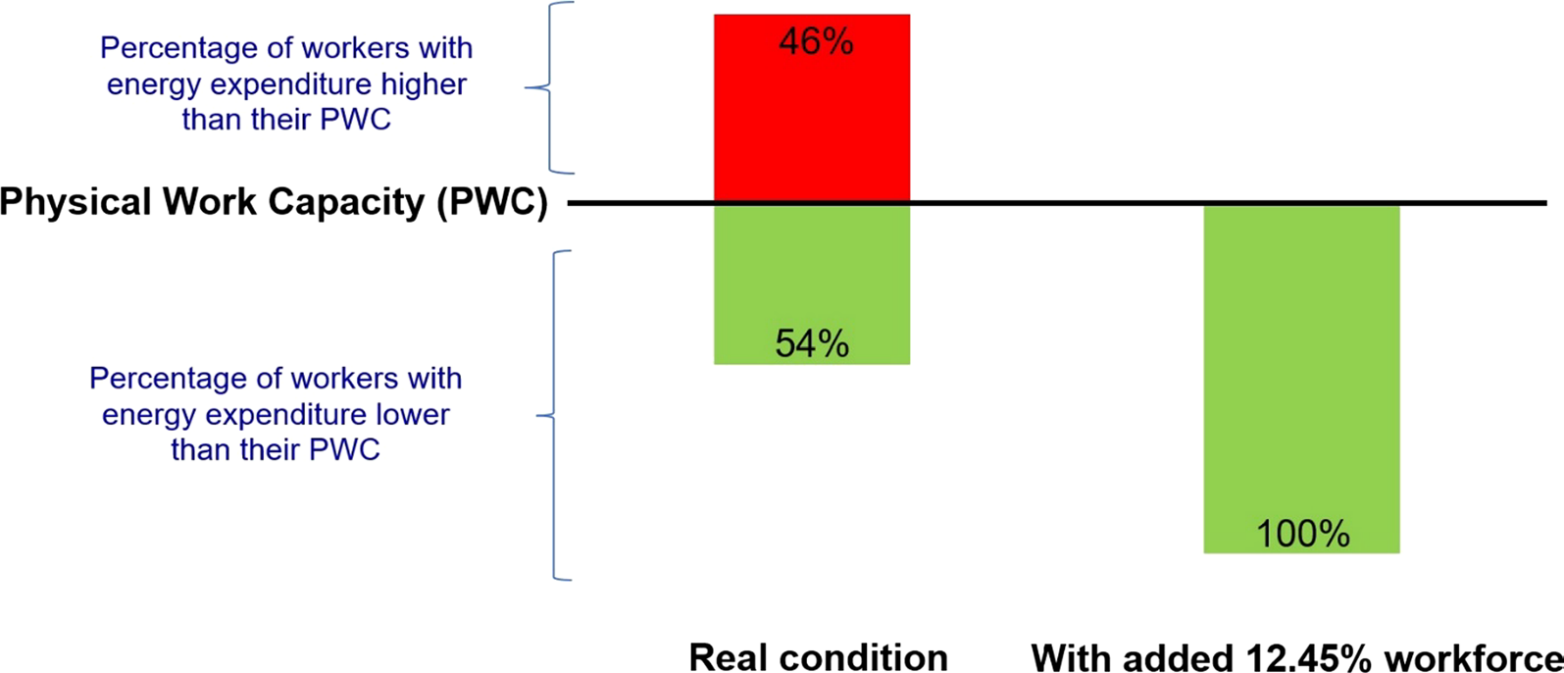
Percentage of workers with energy expenditure higher/lower than their PWC in “real condition” and “condition with added 12.45% workforce”

Figure 1 depicts the percentage of workers with energy expenditure higher/lower than their PWC in two conditions, including “real condition” and “with added 12.45% workforce”.

## Discussion

The findings of the current study among Shiraz municipality cleaners showed that the mean ± standard deviation of VO_2_-max (l min^−1^), PWC (kcal min^−1^), energy expenditure during work (kcal min^−1^), heart rate during work (bpm), RPE 6–20 Borg scale were 2.60 ± 0.66, 4.30 ± 1.09, 4.12 ± 1.02, 96.13 ± 18.09, and 16.60 ± 1.94, respectively.

Up to now, no studies have been carried out on aerobic capacity and PWC of the municipality cleaners in Iran. Therefore, the findings of the current study were compared to those of other studies conducted on other occupations.

The mean VO_2_-max in the present study was close to the values obtained in the research conducted by Tuxworth and Shahnavaz [39] on the Iranian working community to introduce a method for estimating VO_2_-max (2.65 l min^−1^), the study performed by Choobineh et al. [40] on male workers in the industrial sector of Sepidan (2.66 ± 0.35 l min^−1^), and the study conducted by Daneshmandi et al. [18] on male workers in the industrial sector of Shiraz (2.69 ± 0.26 l min^−1^). However, the estimated mean VO_2_-max was significantly lower compared to the values found in the study conducted by Hosseinabadi et al. [17] on workers in the Galvanized section of Semnan Pipe Roll Company (2.88 ± 0.33 l min^−1^), the study performed by Vossoughi [41] on Iranian male students within the age range of 20–25 years (3.03 l min^−1^), the research carried out by Firoozeh et al. [42] on firefighters in Tehran (3.0 ± 0.316 l min^−1^), the study conducted by Khazraee et al. [43] on firefighters in Shiraz (2.79 ± 0.29 l min^−1^), and the study performed by Farhadi et al. [44] on firefighters in Hamadan (3.65 ± 0.56 l min^−1^). Two recent studies have noted that this difference is natural because fitness and high aerobic capacity are important characteristics for selecting firefighters. Therefore, the average aerobic capacity of this group of workers is expected to be higher than that of other occupations. In contrast, the mean VO_2_-max of the present study was higher compared to the results of the study performed by Afshari [45] where the mean VO_2_-max of the students was estimated to be 2.19 ± 0.56 l min^−1^. Given that half of the study population were female, this discrepancy seems reasonable.

With respect to the close proximity of the results of Iranian studies on populations selected from approximately one geographical area [18, 39, 40] and the difference between the present study results and those of the studies conducted in other areas [17, 42, 44] regarding the average aerobic capacity, the difference might be attributed to the body size in different regions. Another reason for this discrepancy might be using different protocols and equipment in the studies. Some studies used the step test according to the Tuxworth-Shahnavaz protocol [39, 40, 42], while some used the Astrand protocols [18, 45]. However, no studies conducted in Iran have used the YMCA protocol used in the present study.

Kuijer et al. [33] performed a study on eight municipality cleaners in the Netherlands and reported that their mean VO_2_-max was 3.7 ± 0.56 l min^−1^, which is significantly higher than the present study. This could be justified by the diversity of the studied populations. Comparison of the findings of Iranian studies to those of European and American countries also showed that the aerobic capacity of the Iranian society was significantly lower in comparison to western societies [17, 18, 45]. However, Preisser et al. [13] indicated that the value of VO_2_-max was 2.46 l min^−1^ for three municipality cleaner groups consisting of two waste collection workers and one group of cleaners. Among the three studied groups, VO_2_-max for cleaners (including five male workers and two female workers) was lower than the two other groups (equal to 2.11 l min^−1^). On the contrary to our expectation, this value was less than that obtained in the present study, which might be associated with the low sample size and different genders of the study participants.

Another objective of the present study was to estimate the amount of energy consumed while working among municipality cleaners in Shiraz. The energy consumed by the cleaners during work was estimated to be 4.12 ± 1.01 kcal min^−1^. Although several studies have been performed on the aerobic capacity of domestic workers [17, 18, 40], no studies have yet been conducted to estimate energy consumption and heart rate during work [11, 13, 32, 46–48]. In a survey conducted by Anjos et al. [49] on Brazilian solid waste collection workers, the average energy consumed during work was estimated to be 5.4 ± 1.4 kcal min^−1^, which is higher than the results of the present investigation.

Heart rate measurement during work was another objective of the present research. The findings showed that the mean heart rate of the cleaners during work was 96.13 ± 18.1 bpm. Thus, considering the work severity, the job of cleaners falls into the middle class [49]. These values were consistent with the heart rates measured in several studies on Dutch solid waste collection workers (heart rates varying between 96 and 99 bpm) [12, 30, 31, 33, 47]. However, they were lower in comparison to the findings of the study conducted by Preisser et al. [13] on Hamburg municipal workers (109.2 ± 12.5 bpm) as well as those of the study performed by Anjos et al. [49] on Brazilian solid waste workers (104 ± 11.7 bpm).

Determining the level of perceived physical exertion using Borg’s RPE 6–20 scale was another objective of the current study. The findings showed that the mean level of physical exertion perceived by the cleaners at work was 16.26 ± 1.94. Therefore, according to Borg’s RPE 6–20 scale, perceived physical exertion was in the hard to very hard range [37]. Despite the ease of use and validation of Borg scale in many countries, due to its subjective nature and the existence of valid physiological indicators, the use of this scale is more limited to laboratory studies, and it is less commonly used in industrial environments. Therefore, this index has been utilized in a few similar studies to assess workload. Indeed, it has only been used with a physiological index in some studies. In a survey carried out by Søgaard et al. [48] on cleaners in Denmark, the average level of perceived physical exertion was 13 (somewhat difficult) [37], which is lower compared to the calculated value in the present investigation. This difference seems to be related to low physical fitness and work conditions and equipment. As mentioned above, the PWC in Iranian society is lower than in Western societies. On the other hand, by interviewing municipality cleaners and also observing the working conditions and equipment, the high physical workload can be attributed to the working conditions (the area that each workforce must clean, the type of surface to be cleaned, the unevenness of the ground, climate, etc.) and non-compliance with ergonomic principles in the design of work equipment.

The present study findings showed no statistically significant relationship between “PWC” and “energy expenditure during work”. It means that there is no balance between the physical workload and PWC among municipal cleaners, which can lead to their physiological fatigue.

The latter is further supported by the fact that a statistically significant interaction was found between PWC and BMI (*P* = 0.045). It could be concluded that the PWC is exceeded due to a lower level of municipality cleaner’s physical fitness.

There was no statistically significant relationship between “energy expenditure during work” with demographic/occupational variables. These findings are not in accordance with Daneshmandi et al. [18], Khazraee et al. [43], and Afshari et al. [45]. The differences between the findings of this study and previous studies can be attributed to the differences in demographic/occupational characteristics of the municipal workers, their lower level of physical fitness, differences in tools and methods for assessing energy expenditure, and different nature of work, and different working conditions.

The ultimate goal of the present study was to evaluate the physical workload of the municipality cleaners by comparing their energy consumption during work to their PWC. The results demonstrated that the physical workloads of 46% of the cleaners were more than their PWCs. In most studies on municipal workers and cleaning workers in different communities, the physical workload was reported to be excessive. For instance, Søgaard et al. surveyed a group of cleaners in Denmark and reported that the physical workload of most participants exceeded the permissible limit [48]. Kemper et al. [31] also performed a study on 23 solid waste workers in the Netherlands and indicated that the physical workload of 39% of the solid waste workers was well above the permitted limit. Several separate studies conducted by Kuijer et al. on waste collection workers in the Netherlands have also shown excessive workloads for this group of workers. In these studies, job rotation was suggested to reduce physical workload. In other words, the workers’ physical workload was decreased significantly after shift rotation [33]. In another study on waste collection workers in Rio de Janeiro, Brazil, Anjos et al. categorized waste collection as an excessively heavy job [49]. It is worth noting that due to the difference between the nature of waste collection and cleaning, these differences seem reasonable.

Comparing the final results of this study to those of two studies carried out in Germany [11, 13] shows that the physical workload was lower in the present study. In a survey conducted by Preisser et al. on 65 municipality cleaners in three separate groups consisting of two separate waste collection workers and one group of Hamburg city cleaners in Germany, the mean oxygen uptake was estimated to be over 30% VO_2_-max in all participants [11, 13]. In the same vein, Frohlich et al. performed a study on cleaners in Hanover, Germany, and concluded that the physical workload of the staff exceeded the permitted level [11].

Based on the present study findings, 12.45% workforce was required to be added to all street cleaners of Shiraz municipality to reduce the physiological workload on the employed workforce. Additionally, given that the physical workload of nearly half of the workers exceeded the permissible limit, solutions such as adjusting the work-rest schedule or using automated equipment to clean the street surface are recommended to reduce the physical workload of this group of workers. Turning shifts among different groups of municipality workers is yet another solution that, if well designed and implemented, can reduce the physical workload of the cleaners.

### Limitations of the study

One limitation of the present study was that the VO_2_-max was calculated using the ergocycle software. There was no inclusion of respiratory parameters like oxygen consumption and carbon dioxide production. This means that the test result is influenced by the variability in the maximum heart rate (MHR) of the individuals. The present study was conducted on municipality cleaners in Shiraz. Thus, the results cannot be generalized to other communities. Indeed, the present study followed a cross-sectional design, and, consequently, the results can be used only for the study time period.

## Conclusions

In conclusion, due to the nature of their work, cleaners are exposed to many occupational hazards and stressors, a significant part of which is attributed to their physical workload. According to the present study results, this task was beyond the PWC for almost half (46%) of the cleaners. Therefore, it is necessary to take control measures to prevent physical exhaustion and injuries among cleaners.

## Data Availability

The datasets used and/or analysed during the current study are available from the corresponding author on reasonable request.

## Abbreviations

YMCA: Young Men’s Christian Association
PWC: Physical Work Capacity
RPE: Rating of Perceived Exertion
EE: Energy Expenditure
BMI: Body Mass Index
